# Formal Volunteering Among Economically Marginalized Older Americans: A Scoping Review

**DOI:** 10.1177/07334648251410281

**Published:** 2026-01-03

**Authors:** Byeongju Ryu, Jihye Baek, Christina Matz, Cal J. Halvorsen

**Affiliations:** 1 Brown School at Washington University in St Louis, St Louis, MO, USA; 2 6019 Boston College School of Social Work, Chestnut Hill, MA, USA; 3 6019 Center on Aging & Work at Boston College, Chestnut Hill, MA, USA; 4 Unit of Occupational Medicine, Karolinska Institute, Stockholm, Sweden

**Keywords:** volunteerism, civic engagement, productive aging, successful aging, social inclusion

## Abstract

Volunteering in later life has been widely recognized as a pathway to support healthy aging. However, economically marginalized older adults often face barriers to participating in formal or organizational volunteering. We aimed to identify the associated factors and outcomes of formal volunteering among older adults with low income or wealth in the U.S. Following PRISMA guidelines, we systematically searched five databases for peer-reviewed empirical studies published since 2005. Guided by the ecological framework of the antecedents and consequences of productive engagement in later life, we summarized factors and outcomes associated with formal volunteering at the individual, community, and societal levels. Older adults with lower economic status often experience disproportionately greater benefits from volunteering but face barriers to participation. Addressing these barriers could promote equitable access, enhance volunteer inclusion, and foster broader societal benefits.

**What This Paper Adds**

• This scoping review adds to the literature on formal volunteering among older adults with low economic status.
• The review advances understanding of the relationship between formal volunteering among older adults with low economic status and factors at individual, community, and societal levels.
• Identification of gaps in the literature offers implications for future research on this topic.

**Applications of Study Findings**

• This study highlights the importance of community, institutional, and policy supports, such as transportation services, stipends, and flexible scheduling, in promoting inclusion in formal volunteering among socioeconomically diverse older adults.
• Future research may explore how the inclusion of socioeconomically diverse older adults in formal volunteering influences broader communities and society.

## Introduction

Approximately one-quarter of adults aged 55 and over participate in formal volunteering in the U.S. (AmeriCorps, 2023). Formal volunteering, defined as unpaid (or minimally compensated to offset costs) participation in organized activities or programs facilitated by established nonprofit organizations (Morrow-Howell et al., 2009), may support healthy aging. Volunteering is associated with improved health and well-being outcomes, including self-reported health, blood pressure, epigenetic aging, functional independence, mortality, cognitive function, depressive symptoms, life satisfaction, and self-efficacy (Anderson et al., 2014; Kim et al., 2023, 2025).

However, access to volunteering opportunities is not evenly distributed. Volunteers often have higher incomes, more education, better health, and stronger social networks, factors that make it easier to find and participate in volunteer opportunities (Gonzales et al., 2015). Volunteering may be difficult to access for older adults who lack financial resources, serve as caregivers, or live with certain disabilities, circumstances that are more prevalent among those with lower wealth and income (McBride, 2007; Tang et al., 2009). Barriers may include little knowledge about volunteering opportunities, costs of volunteering, lower health, and lack of transportation and prior volunteer experience (Carr et al., 2015). Older adults with low socioeconomic status may also disengage from educational institutions earlier, which serves as a volunteer recruitment channel (Park & Morrow-Howell, 2020). However, research shows that older adults from socioeconomically disadvantaged backgrounds spend considerable time helping neighbors, friends, or others outside their households through personal networks rather than organizations (Reyes, 2024). This “informal volunteering” may reflect a spectrum of motivations, from feeling a sense of obligation (especially when the recipient is known) to altruistic reasons (Nakamura et al., 2024).

Scholars have emphasized the importance of engaging socioeconomically disadvantaged older adults in volunteerism (Cruikshank, 2013; Moody, 2001; Morrow-Howell et al., 2015; Park & Morrow-Howell, 2020). Several national-level initiatives in the U.S., such as the federally supported Senior Companion Program and AARP’s Experience Corps, are known for recruiting many low-income older volunteers and providing modest stipends to help offset costs, such as for transportation to the volunteer site (Carr et al., 2015).

Research suggests that older individuals with fewer resources often experience disproportionately greater benefits from volunteering (Carr et al., 2015; Morrow-Howell et al., 2009). Volunteering may help low-income older adults build and strengthen social connections and skills while contributing to community well-being (Benenson & Stagg, 2016; McBride et al., 2006). Removing barriers to participation could lead to higher volunteer engagement and generate broader public health and societal benefits, including improved health outcomes, reduced dependence on social insurance programs, increased contributions to the economy, stronger retirement security, and a thriving nonprofit sector (Gonzales et al., 2015; Morrow-Howell & Greenfield, 2016). Given these benefits, older adults who wish to participate in formal volunteering should be supported in doing so (Martinson & Minkler, 2006). To inform future research and guide policy and program developments that facilitate volunteer opportunities for socioeconomically marginalized older adults, a comprehensive review of the literature is needed.

## Research Aims

We aimed to review U.S.-based empirical studies on formal volunteering among older adults of low economic status, with a particular emphasis on understanding the individual, community, and societal factors and outcomes related to their participation. We focused specifically on economic status, which reflects the cumulative effects of educational attainment, employment opportunities, and resource access throughout the life course, often indicated through measures of income and wealth (Congressional Research Service, 2021; Duncan et al., 2002), due to its association with volunteering behaviors in later life. We focused on the U.S. context because of its unique history of volunteerism and mix of federally funded and nonprofit volunteer programs (Chambré, 2020).

We also explored study characteristics, designs, analytical approaches, and theoretical or conceptual frameworks in the literature to capture the breadth of current knowledge and identify research gaps. We conducted a scoping review instead of a systematic review: While systematic reviews typically address narrower research questions, such as evaluating the effectiveness of a particular intervention, a scoping review aims to identify and map the breadth of evidence available on a given topic and is often used to inform future research (Pollock et al., 2024).

## Guiding Conceptual Framework

Given that formal volunteering is part of the broader concept of productive aging (Park & Morrow-Howell, 2020), we employed an ecological framework of the antecedents and consequences of productive engagement in later life (Figure 1; Morrow-Howell & Greenfield, 2016). This framework, grounded in the ecological systems theory (Bronfenbrenner, 1993), views individual behaviors and well-being as factors that are shaped by dynamic interactions with personal attributes and the surrounding environment. In our study, this framework was operationalized to organize and interpret findings across three nested levels: individual, community, and societal.

**Figure 1.**
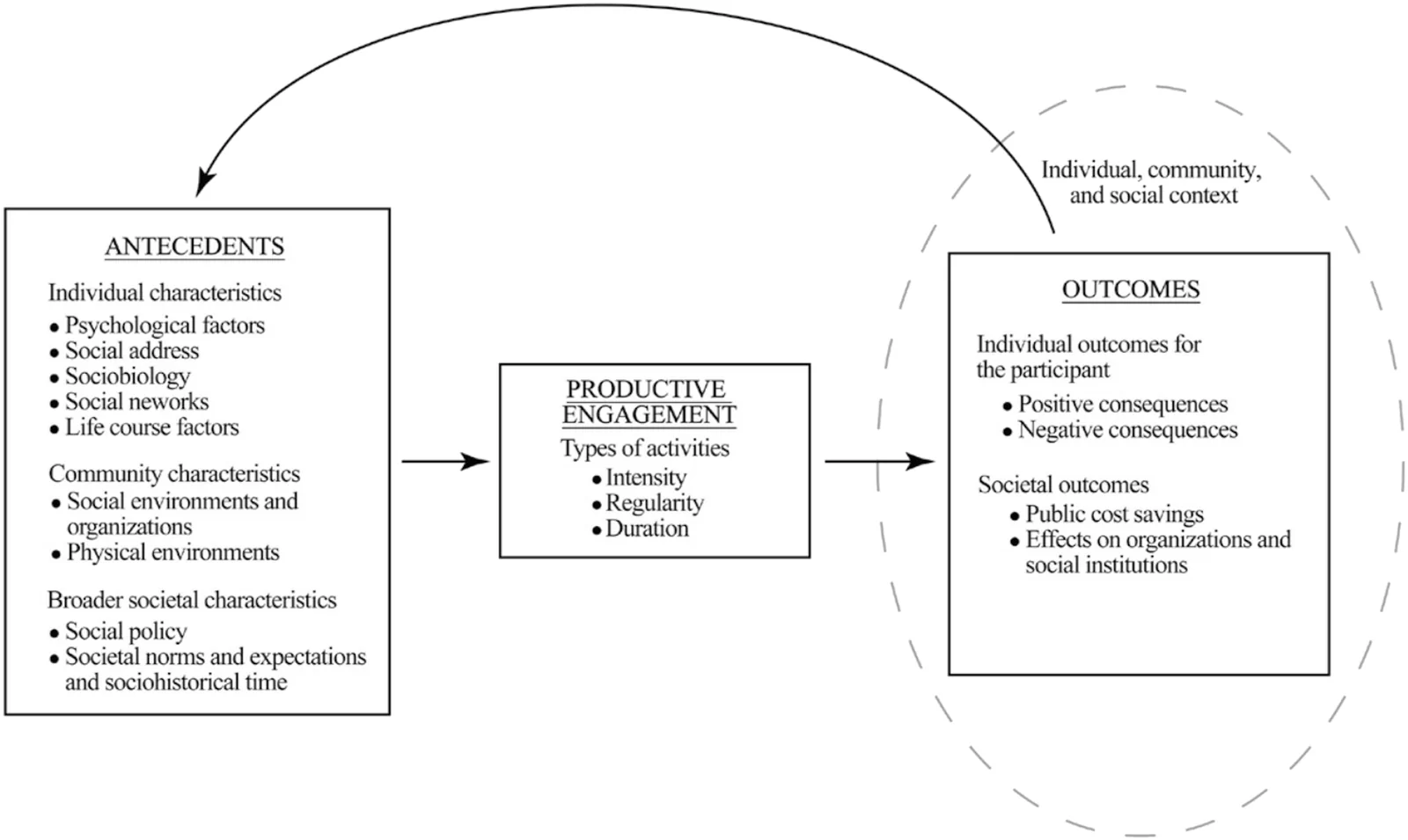
The ecological framework of the antecedents and consequences of productive engagement in later life. *Note*. Reprinted from handbook of aging and the social sciences, 8th edition (p. 300), by Morrow-Howell and Greenfield (2016), with permission from Elsevier

Individual characteristics refer to attributes specific to a person or group, such as personal values related to helping others. Community characteristics encompass contextual factors tied to specific localities or institutions, such as efforts by organizations to recruit older volunteers. Societal characteristics involve broader influences that extend beyond individual communities, including social policies and cultural norms related to productive activity in later life. To uphold parsimony, we conceptualize linkages as primarily unidirectional, although many relationships are likely bidirectional. The dotted oval and bidirectional arrows in Figure 1 represent reciprocity, such as when the benefits older adults gain through volunteering may, over time, strengthen their motivation and capacity to engage again, thereby reinforcing the antecedent conditions that foster continued participation.

## Methods

This scoping review followed PRISMA guidelines (Supplement Appendix A) and employed a five-stage scoping review methodology (Arksey & O'Malley, 2005). These included defining research questions; identifying relevant studies; selecting studies; charting the data; and collating, summarizing, and reporting results.

### Defining Research Questions

Considering older adults with low economic status in the U.S., we examined two research questions: (1) What factors are associated with participation in formal volunteering? and (2) What are the outcomes related to formal volunteering?

### Identifying Relevant Studies

We conducted a literature search on May 1, 2024, across five search engines: Scopus, EBSCO, ProQuest, PubMed, and Medline. We identified search terms related to “formal volunteering” and “economically marginalized older adults” through preliminary searches. Terms within each concept were combined using the Boolean operator “OR,” and the two main concepts were linked with “AND” (Table 1).

**Table 1.** Search Keywords (Title, Abstract, and Keywords)

“Volunteering” keywords	“Population” keywords
“Volunteer*” AND (“product*” OR “active*” OR “experience*” OR “senior*” OR “ameri*” OR “corp*” OR “cncs*” OR “serv*” OR “scor*” OR “retire*” OR “rsvp*” OR “foster*” OR “fgp*” OR “companion*” OR “scp*” OR “civic*” OR “cogen*” OR “encor*” OR “aarp*” OR “engag*” OR “partici*”)	(“Older-adult*” OR “older-people*” OR “older-person*” OR “older-individual*” OR “aging*” OR “ageing*” OR “elder*” OR “senior*” OR “ageis*”) AND (“vulner*” OR “marginal*” OR “disadvant*” OR “income*” OR “asset*” OR “resource*” OR “econom*” OR “financ*” OR “povert*” OR “medicaid*”)

### Study Selection

Studies were selected based on multiple inclusion criteria. First, to capture recent research, we included only studies published since 2005, when the White House Conference on Aging (WHCoA) highlighted civic participation as an important focus of U.S. aging policy (WHCoA, 2005). Second, we included empirical studies published in peer-reviewed journals to ensure the rigor of study findings, excluding non-peer-reviewed sources, grey literature, dissertations, and abstracts. Third, the studies had to examine formal volunteering in later life, clearly describing volunteer work carried out through organizations. Informal helping or political forms of civic participation were excluded from this review.

Fourth, at least half of study participants had to be aged 50 or older, reflecting age requirements for major nonprofit (e.g., Experience Corps, minimum volunteer age: 50) and federal (e.g., AmeriCorps Seniors, minimum volunteer age: 55) programs. When exact participant age data were not provided, studies were included if they explicitly stated that their population of interest was older adults.

Lastly, the studies had to focus on economically marginalized older adults, which we operationalized as having low income or wealth. We included studies whose specific aims, hypotheses, or research questions focused on older adults with lower economic status or examined differences in factors or outcomes associated with volunteering across economic strata. We did not impose a uniform economic threshold, such as the Federal Poverty Line, because definitions varied across studies. Instead, we included studies that clearly described their participants as having low economic status, using terms such as “low income,” “low wealth,” or “economically marginalized” or “disadvantaged,” given that specific quantitative cutoffs were often unavailable or inconsistently reported.

The study selection process involved an initial screening of titles and abstracts, followed by a full-text review. We imported all citations into the Zotero reference management software, removing duplicates. The first and second authors independently screened and reviewed each article, addressing discrepancies through consensus. The search results and study selection process are documented in Figure 2 (Levac et al., 2010).

**Figure 2.**
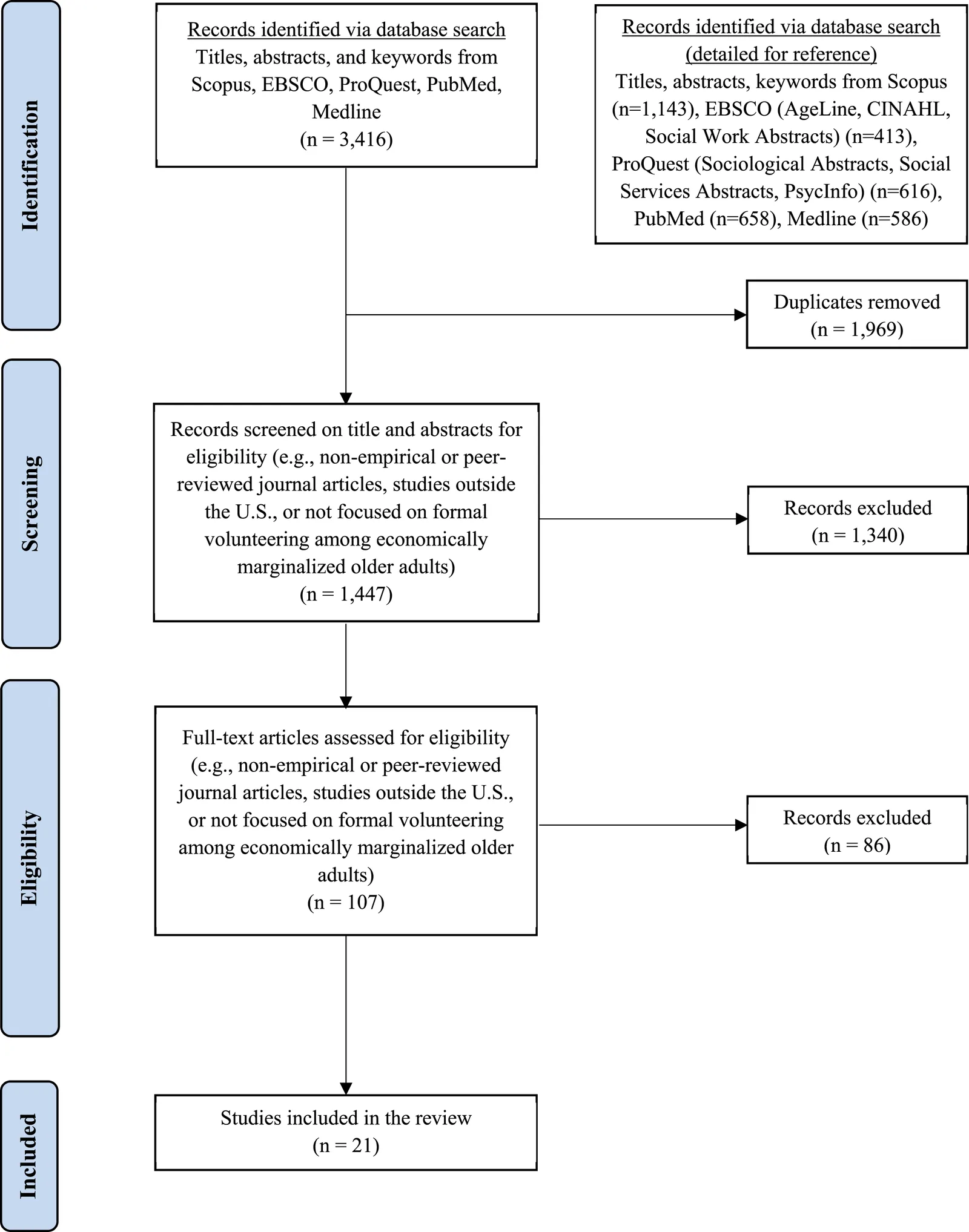
Preferred reporting items for systematic reviews and meta-analyses (PRISMA) diagram (Moher et al., 2009)

### Charting the Data

Studies that met inclusion criteria were extracted into a Microsoft Excel file. The database contained author names, year of publication, title, study objectives, guiding theoretical or conceptual frameworks, participant characteristics, study design, data sources, analytic approaches, descriptions of volunteer programs, and associated factors and outcomes of formal volunteering.

### Collating, Summarizing, and Reporting Results

A descriptive summary analysis was performed to identify patterns in sample characteristics, study designs, analytical methods, and theoretical frameworks. The details of formal volunteering programs, along with associated factors and outcomes, were then summarized and reported. Drawing on the ecological framework of the antecedents and consequences of productive engagement in later life (Figure 1; Morrow-Howell & Greenfield, 2016), we conducted a thematic analysis of factors and outcomes associated with formal volunteering. The first and second authors independently identified preliminary themes within each domain of the framework, compared their results, and addressed discrepancies through iterative discussions that led to consensus. In this dynamic approach, no inter-rater reliability was calculated (Gottlieb et al., 2021). These refined themes were then reviewed and finalized through discussions with the third and fourth authors, resulting in full consensus on the final set of themes.

## Results

### Descriptive Summary

#### Study Characteristics

This review included 21 articles, encompassing 14 quantitative, four qualitative, and three mixed-methods studies. Table 2 presents an overview of these articles; Supplement Appendix B presents each article’s study objectives, participant characteristics, study design and analytic approach, data source, and theoretical frameworks; and Supplement Appendix C presents descriptions of the volunteer programs highlighted in each article as well as the associated factors and outcomes of formal volunteering among economically marginalized older adults.

**Table 2.** Overview of Included Studies (*N* = 21)

#	Authors (year)	Program name	Analytic approach	Sample eligibility or comparison criteria by economic status
1	Butler (2006)	Senior Companion Program	Mixed-method and cross-sectional	All participants ≤125% FPL
2	Cao et al. (2021)	Senior Companion Program	Qualitative	Predominantly low income (no threshold reported)
3	Cao et al. (2023a)	Senior Companion Program	Mixed-method and cross-sectional	Predominantly low income (no threshold reported)
4	Cao et al., (2023b)	Senior Companion Program	Quantitative and cross-sectional	Predominantly low income (no threshold reported)
5	Carlson et al. (2009)	Experience Corps Program	Quantitative and pre-and post-test	Predominantly low income (no threshold reported)
6	Cho et al. (2018)	Not specific to a single program	Quantitative and cross-sectional	Compared participant groups using FPL threshold
7	Cho et al. (2020)	Not specific to a single program	Quantitative and cross-sectional	Compared participant groups using FPL threshold
8	Clark-Ibáñez et al. (2021)	Cultivando Sabiduría	Mixed-method and longitudinal	Predominantly low income (no threshold reported)
9	Fields et al. (2023)	The Caring Callers Program	Qualitative	Predominantly low income (no threshold reported)
10	Hood et al. (2018)	Senior Companion Program	Qualitative	62.7% of participants had monthly income < FPL
11	Kim & Halvorsen. (2021)	Not specific to a single program	Quantitative, longitudinal, and quasi-experimental	Compared lowest vs highest wealth quintiles
12	Martinez et al. (2011)	Not specific to a single program	Qualitative	71% of participants had annual income < $15,000
13	McBride et al. (2011)	Experience Corps Program	Quantitative and pre-and post-test	Compared stipended (lower income) vs non-stipended (higher income) volunteers
14	Moon (2012)	Not specific to a single program	Quantitative and cross-sectional	79.1% of participants had annual income < $20,000
15	Morrow-Howell et al. (2009)	Not specific to a single program	Quantitative and cross-sectional	Compared across annual income strata
16	Park et al. (2017)	Not specific to a single program	Quantitative and cross-sectional	All participants ≤185% FPL
17	Tan et al. (2006)	Experience Corps Program	Quantitative and randomized controlled trial	69% of participants had annual income < $15,000
18	Tan et al. (2009)	Experience Corps Program	Quantitative and longitudinal	53.4–60.5% of participants had annual income < $15,000
19	Tang et al. (2009)	Not specific to a single program	Quantitative and cross-sectional	Compared across annual income strata
20	Tang et al. (2010a)	Not specific to a single program	Quantitative and longitudinal	Compared across annual income strata
21	Tang et al. (2010b)	Not specific to a single program	Quantitative and longitudinal	Compared across socioeconomic strata (combined income and education measure)

*Note.* FPL = federal poverty line.

Sample sizes ranged from 17 to 22,958 in quantitative studies, 18 to 70 in qualitative studies, and 66 to 177 in mixed-methods studies. Most quantitative studies analyzed survey data, except one examining neuroimaging data (Carlson et al., 2009). Most qualitative studies gathered data through focus groups or individual interviews, while one mixed-methods study (Clark-Ibáñez et al., 2021) further analyzed data from diverse sources, including photographs and oral histories. Among the quantitative studies, seven employed a cross-sectional design (Cao et al., 2023b; Cho et al., 2018, 2020; Moon, 2012; Morrow-Howell et al., 2009; Park et al., 2017; Tang et al., 2009), while seven utilized longitudinal designs, including experimental (Tan et al., 2006), quasi-experimental (Kim & Halvorsen, 2021), correlational (Tan et al., 2009; Tang et al., 2010a, 2010b), and pre-post designs (Carlson et al., 2009; McBride et al., 2011). Two mixed-methods studies used a cross-sectional design (Butler, 2006; Cao et al., 2023a), and one adopted a longitudinal approach (Clark-Ibáñez et al., 2021).

We identified 15 studies guided by theoretical and conceptual frameworks. These include two (Butler, 2006; Cho et al., 2018) that drew on role theory (e.g., Adelmann, 1994). Another three (Tang et al., 2009; 2010a; 2010b) were guided by an institutional perspective (e.g., Sherraden et al., 2001). The social model for health promotion (Fried et al., 2004) was used in two studies (Tan et al., 2006, 2009). The remaining eight studies varied in their use of guiding theoretical or conceptual frameworks.

#### Participant Characteristics

Sixteen studies with primary data recruited their participants from specific volunteer programs, including the Senior Companion Program (Butler, 2006; Cao et al., 2021, 2023a, 2023b; Fields et al., 2023; Hood et al., 2018), Experience Corps (Carlson et al., 2009; McBride et al., 2011; Tan et al., 2006, 2009), and Cultivando Sabiduría (Clark-Ibáñez et al., 2021). Four studies with primary data recruited participants from multiple programs (Morrow-Howell et al., 2009; Tang et al., 2009, 2010a, 2010b). Two studies with primary data identified eligible participants in specific geographic locations (Martinez et al., 2011; Moon, 2012). Further, four studies used secondary data from the Health and Retirement Study (Cho et al., 2018, 2020; Kim & Halvorsen, 2021; Park et al., 2017).

Most studies assessed participants’ economic status using individual or household income, although one study (Kim & Halvorsen, 2021) focused on wealth. Thirteen studies described participants as predominantly low income, including six that did not report detailed income distributions (Cao et al., 2021, 2023a, 2023b; Carlson et al., 2009; Clark-Ibáñez et al., 2021; Fields et al., 2023). Among the seven studies that provided participants’ income distributions, two restricted their samples to individuals below 125% (Butler, 2006) or 185% (Park et al., 2017) of the Federal Poverty Line (FPL). The remaining five reported the proportions of participants below the FPL or at or below specific annual earnings amounts (e.g., $15,000; Hood et al., 2018; Martinez et al., 2011; Moon, 2012; Tan et al., 2006, 2009).

Eight studies examined older adults across diverse economic strata, comparing participants with lower economic status to their more affluent counterparts in examining outcomes of interest. Cho et al. (2018, 2020) used the FPL as a threshold to classify income groups. McBride et al. (2011) compared lower-income stipended to higher-income non-stipended volunteers. Morrow-Howell et al. (2009) and Tang et al. (2009, 2010a, 2010b) compared lower- and higher-income volunteers without specifying a particular threshold, and Kim and Halvorsen (2021) compared those in the lowest and highest wealth quintiles.

The demographic characteristics of study participants are presented in Supplement Appendix B. 17 studies reported the mean age of participants (range: 65–77 years). In the 18 studies reporting participants’ gender, women were always in the majority (range: 53%–100%). Among the 18 studies reporting participants’ educational attainment, 10 indicated that most participants had a high school education or less, while eight studies reported that most participants had more than a high school education. Most studies included predominantly White participants (10 studies), followed by African American (8 studies) and Latinx (1 study) participants. None of the studies reported Asian, American Indian, Alaska Native, Native Hawaiian, or Pacific Islander groups as the majority of participants.

### Thematic Results

Our analysis identified eight themes across individual, community, and broader societal characteristics of formal volunteering among economically marginalized older adults. Seven themes emerged related to outcomes of formal volunteering at an individual or societal level (Figure 3). In-depth descriptions of these thematic results are provided in Tables 3 and 4.

**Figure 3.**
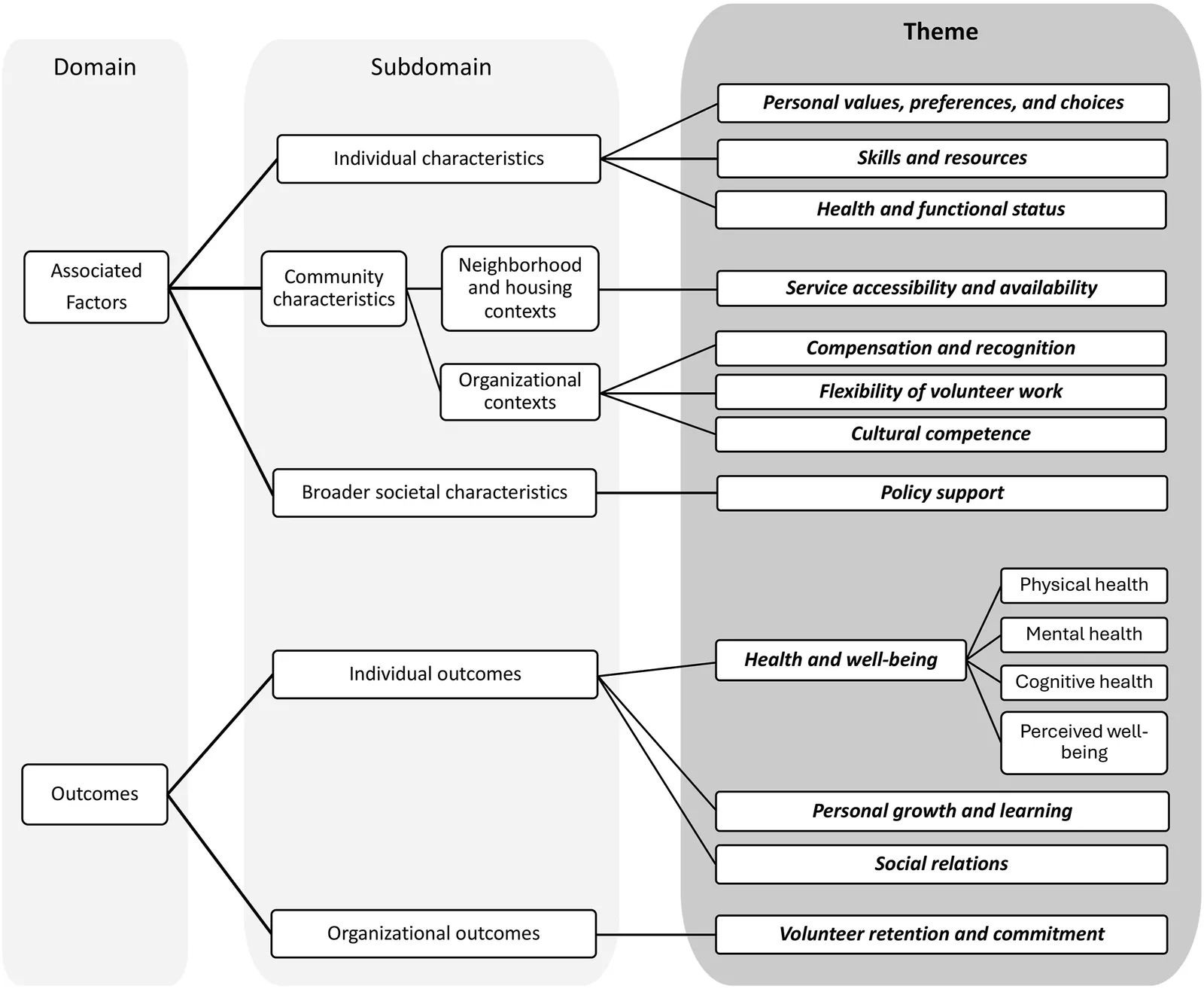
Themes emerging from factors and outcomes associated with formal volunteering among economically marginalized older adults in the US

**Table 3.** Thematic results of factors associated with formal volunteering

Domain	Subdomain		Theme	Characteristic	Study^a^
Associated Factors	**Individual characteristics**		Personal values, preferences, and choices	Personal values for helping others; A desire to make their communities a better place	#8, 9
				A desire to learn a new skill	#8
				Being well-matched with clients who have similar interests; Preferences of socializing with peer volunteers with the same backgrounds	#3, 9
				Post-retirement work	#6
			Skills and resources	Perception of usefulness and easiness of information communication and technologies	#7
				English proficiency	#8
				Personal resources (e.g., money and time)	#2, 12
			Health and functional status	Health and medical needs	#12, #20
	**Community characteristics**	*Neighborhood and housing contexts*	Service accessibility and availability	Transportation; Geographic accessibility	#2, 12
				Personal asks for being a volunteer; Publicizing opportunities to participate in volunteer activities	#12
				Community resources and information for volunteers who are family caregivers	#2
				Senior housing residency	#16
		*Organizational contexts*	Compensation and recognition	Provision of stipend	#1, 13, 19
				Transportation (e.g., mileage reimbursement)	#2, 12, 19
				Treatment by the staff; Recognition from the volunteer organization; Ongoing support and adequate training	#12, 19, 21
			Flexibility of volunteer work	Flexible and reasonable role/time commitments	#12, 19, 21
			Cultural competence	Linguistically and culturally relevant program and curriculum	#8
				Organizational resources and commitment for cross-cultural volunteering	#3
	**Broader societal characteristics**		Policy support	Program availability and support (e.g., federally subsidized programs and programs across the states operated by national-level nonprofit organizations)	#1, 2, 3, 4, 5, 9, 10, 13, 15, 17, 18, 19

^a^The study numbers correspond to those listed in Table 2.

**Table 4.** Thematic results of outcomes associated with formal volunteering

Domain	Subdomain	Theme	Characteristic		Study^a^
Outcomes	**Individual outcomes**	Health and well-being	Physical health	Increased physical activity (e.g., increased mean minutes and kilocalories per week)	#17, 18
				Increased self-reported health	#11
			Mental health	Fewer depressive symptoms	#1, 10, 11
				Relieved stress	#2
			Cognitive health	Improved executive functions and activities in brain regions	#5
			Perceived well-being	Preventing the negative reinforcement of loneliness; Offsetting a feeling of loneliness associated with social distancing in the COVID-19	#4, 9
				Changes in subjective quality of life and life satisfaction; Increased a sense of purposeful life, accomplishment, purposeful use of time, and independence	#1, 3, 8, 9, 10, 12, 13, 14, 15, 21
		Personal growth and learning	Gaining a new perspective (e.g., new perspective on aging)		#9, 10
			Learning new skills and information; Training		#2, 8, 9, 10
		Social relations	Increased social interaction; Expanded social networks; Decreased social isolation; Increased social support		#1, 2, 3, 4, 8, 9, 10, 12, 13
	**Organizational outcomes**	Volunteer retention and commitment	Changes in volunteer retention, turnover, and time commitments		#9, 13, 20, 21

^a^The study numbers correspond to those listed in Table 2.

#### Factors Associated with Formal Volunteering Among Economically Marginalized Older Adults

Eighteen studies explored the factors related to participation in formal volunteering of older adults with low economic status across individual, community, and broader societal levels (Table 3).

##### Individual Characteristics

Eight studies examined individual-level factors. We identified three themes: personal values, preferences, and choices; skills and resources; and health and functional status.

##### Personal Values, Preferences, and Choices

A personal desire to help others, make the community a better place, or learn a new skill was identified as a facilitator of volunteering among low-income Latinx older volunteers in Cultivando Sabiduría, who assisted teachers and students at school, provided childcare at church, and distributed food in mobile pantries (Clark-Ibáñez et al., 2021). The personal value for helping others was reported as the facilitator among low-income older volunteers in a telephone-based intervention program where volunteers provided weekly calls to socially isolated peers during the COVID-19 pandemic (Fields et al., 2023).

Being matched with clients who had similar interests or life experiences was also mentioned as a factor for greater satisfaction among volunteers in the program. In a multicultural setting, preferences for socializing with peer volunteers from similar backgrounds (e.g., same gender, country, or language) acted as a barrier to interacting with volunteers from different backgrounds in the Senior Companion Program (Cao et al., 2023a). Using a national sample from the Health and Retirement Study (HRS), Cho et al. (2018) found that among retirees near the federal poverty level, those who engaged in post-retirement paid work spent more time volunteering than those who did not.

##### Skills and Resource

Individuals’ skills and resources, including language, technology, money, and time, were found to influence volunteer engagement. Clark-Ibáñez et al. (2021) reported that limited English proficiency among older Latinx volunteers was a barrier to volunteering. Lower-income participants in the HRS were more likely to volunteer if they perceived technology as being useful and easy to use (Cho et al., 2020). In focus group interviews with older adults in Baltimore, Martinez et al. (2011) found that respondents noted difficulty in volunteering when it conflicted with other roles (e.g., paid employment). Similarly, Cao et al. (2021) revealed that volunteers often felt overwhelmed and unable to commit to volunteer roles due to conflicts with other community responsibilities, such as helping neighbors.

##### Health and Functional Status

Across 13 volunteer programs in the U.S., lower-income volunteers with poorer health were more likely to stop volunteering than their more affluent and healthier counterparts (Tang et al., 2010a). Similarly, low-income older adults reported in focus groups that they had difficulty volunteering due to functional limitations, including limited mobility or vision loss (Martinez et al., 2011).

##### Community Characteristics

Nine studies identified community-level factors influencing participation in volunteering. Themes included service accessibility and availability within neighborhood and housing environments, compensation and recognition, cultural competence, and flexibility of volunteer work in organizational contexts.

##### Service Accessibility and Availability

Three studies underscored the importance of community resources in facilitating formal volunteering among low-income older adults. Public transportation was a key factor, with Martinez et al. (2011) finding that accessible transit options to volunteer organizations or accessible activity locations could enhance participation. Cao et al. (2021) noted that for certain ethnic enclaves, a lack of transportation from their communities to volunteer sites limited participation.

Access to information about community resources played a significant role in volunteer participation. For family caregivers, whose volunteering was often constrained by caregiving responsibilities, providing information on available resources could facilitate volunteering (Cao et al., 2021). Knowing about volunteer opportunities, including through personal asks or publicized opportunities, was also important for fostering the inclusion of low-income volunteers (Cao et al., 2021).

Senior housing—non-institutional, congregate housing for older adults—could facilitate formal volunteering among low-income older adults. One study using HRS data found that low-income older adults in senior housing were more likely to participate in formal volunteering than those with similar characteristics residing in conventional homes (Park et al., 2017).

##### Compensation and Recognition

In organizational contexts, material incentives such as stipends and mileage reimbursement were shown to facilitate volunteering among low-income older adults. Across 13 volunteer programs in the U.S., lower-income volunteers perceived receiving a stipend as an important return for their service (Tang et al., 2009). Low-income volunteers in the Senior Companion Program (Butler, 2006) reported that stipends were essential for meeting their basic needs. Analyzing Experience Corps participant data across 18 sites, McBride et al. (2011) suggested that stipends could enhance the inclusion of ethnic minorities and low-income individuals while increasing retention.

Low-income older volunteers in the Senior Companion Program reported challenges with transportation support, such as inadequate assistance for car maintenance and limited mileage reimbursement (Cao et al., 2021). Providing free parking passes and transportation from volunteers’ residences to activity sites was reported as a way to facilitate participation (Martinez et al., 2011; Tang et al., 2009).

How staff and volunteer organizations treated and recognized volunteers was noted as an important consideration. In a study of 10 volunteer programs, ongoing support and adequate training were associated with greater volunteer time commitment (Tang et al., 2010b). In the same project, recognition from the volunteer organization was identified as a key institutional facilitator (Tang et al., 2010a). Another study found that being treated respectfully and having their contributions acknowledged through rewards, certificates, or records that could be beneficial for future employment, enhanced volunteer engagement, whereas negative experiences, such as feeling undervalued, discouraged their involvement (Martinez et al., 2011).

##### Flexibility of Volunteer Work

A structured volunteer routine helped older volunteers in the Senior Companion Program avoid sedentary, isolated lives (Butler, 2006). However, continued engagement could be challenging without flexible and reasonable time commitments. The concern about committing to overly structured roles—such as fixed schedules with limited flexibility—was reported as unattractive to lower-income older volunteers when they conflicted with other priority roles, such as employment (Martinez et al., 2011). Volunteers reported that the ability to choose their volunteer activities and set their own schedules was important for sustaining their engagement (Tang et al., 2009) and increased their weekly time commitment to volunteering (Tang et al., 2010b).

##### Cultural Competence

The cultural competence of volunteer organizations was emphasized as a crucial factor in fostering the inclusion of diverse older volunteers. In a study on older Latinx volunteers, having a linguistically and culturally relevant program—such as ensuring that most participants or staff shared the same ethnic background or were bilingual—facilitated their engagement (Clark-Ibáñez et al., 2021). When the program’s objective was to foster cross-cultural volunteering experiences, intentional efforts by the organizations were needed, including the provision of translation services and training and events within a multicultural setting that encouraged cross-cultural engagement (Cao et al., 2023a).

##### Broader Societal Characteristics

Policy support emerged as a broader societal factor in volunteering. 12 studies recruited older volunteers below specific income thresholds from federally subsidized programs (e.g., the Senior Companion Program) or nationwide programs operated by national nonprofit organizations (e.g., Experience Corps). These programs, funded by public or charitable resources, have actively sought to engage economically marginalized older adults through various forms of support (e.g., financial incentives for those who meet income criteria).

#### Outcomes of Formal Volunteering Among Economically Marginalized Older Adults

Sixteen studies identified outcomes of formal volunteering among older adults with low economic status at the individual or organizational levels (Table 4).

##### Individual-Level Outcomes

Three themes emerged across 16 studies: health and well-being, personal growth and learning, and social relations.

##### Health and Well-Being

Two studies, which investigated low-income older volunteers with continuous participation in Experience Corps, revealed that the program led to increases in participants’ physical activity levels in both the shorter term (after 4 to 8 months; Tan et al., 2006) and longer term (after 3 years; Tan et al., 2009). Employing a quasi-experimental design using the HRS, a study found that older volunteers in the lowest wealth quintile experienced significantly higher gains in self-reported health than their wealthier counterparts (Kim & Halvorsen, 2021).

Older volunteers also experienced mental health benefits. Volunteers in the Senior Companion Program reported lower average depression scores than the cutoff for elevated depression (Butler, 2006). Another study found that Senior Companion volunteers could reduce depressive symptoms through increased social interaction (Hood et al., 2018). One study noted that participants in the program experienced stress relief through social interaction with clients, such as talking, playing games, and sharing cultural similarities (Cao et al., 2021). A quasi-experimental study using the HRS found that volunteering alleviated depressive symptoms among volunteers, regardless of their wealth status (Kim & Halvorsen, 2021).

Another potential benefit was improvement in cognitive health. One study found that Experience Corps participants exhibited enhanced cognitive functions, including increased brain activity and improved executive function, suggesting that the program might mitigate age-related cognitive and neural decline (Carlson et al., 2009).

Older volunteers reported perceived well-being benefits, such as decreased loneliness (Cao et al., 2023b; Fields et al., 2023), a greater sense of meaning and achievement in life (Cao et al., 2023a; Hood et al., 2018; Morrow-Howell et al., 2009; Tang et al., 2010b), feelings of satisfaction or reward in their roles (Butler, 2006; Clark-Ibáñez et al., 2021; Martinez et al., 2011), and more purposeful or productive use of their time (Fields et al., 2023; McBride et al., 2011). Furthermore, lower-income volunteers felt more empowered and valued when engaging in volunteer tasks that demanded expertise, as opposed to those requiring less skill (Morrow-Howell et al., 2009). One study noted no significant association between volunteer status and self-rated quality of life or life satisfaction (Moon, 2012).

##### Personal Growth and Learning

Older volunteers reported that their experiences contributed to their personal growth and learning. Senior Companion volunteers gained a deeper understanding of aging through their clients’ experiences, which helped them better prepare for their own aging (Hood et al., 2018). Many applied lessons learned from conversations with clients to their own lives (Fields et al., 2023). Some noted that they acquired new skills from training sessions (Clark-Ibáñez et al., 2021), revisited skills they had not used in years during their volunteer services (Fields et al., 2023), and received valuable information through monthly training sessions, such as transportation and community resources available to them (Cao et al., 2021).

##### Social Relations

Participants reported multiple social benefits from their volunteer work, including broader social networks, enhanced social interaction and support, and reduced social isolation. Older volunteers in the Senior Companion Program developed friendships with peer volunteers and deepened relationships with clients (Cao et al., 2023a; Hood et al., 2018), noting the feeling of companionship through social interactions (Butler, 2006). Similarly, volunteers in the Experience Corps program reported an increase in friends and acquaintances (McBride et al., 2011). Volunteers in other programs also experienced social benefits, such as increased social interaction (Martinez et al., 2011) and a stronger sense of social connectedness with people outside their homes (Clark-Ibáñez et al., 2021). During the COVID-19 pandemic, telephone-based volunteering alleviated volunteers’ own feelings of isolation caused by social distancing (Fields et al., 2023).

A study on ethnically diverse older volunteers found greater peer support and social benefits when they were connected with others who shared their languages and cultural backgrounds (Cao et al., 2021). Conversely, in a cross-cultural volunteer program, volunteers could enhance their social networks by interacting with peers from diverse demographic or socioeconomic backgrounds during orientations and training sessions (Cao et al., 2023a).

##### Organizational Outcomes

Four studies focused on volunteer retention as an organizational outcome of formal volunteering. Lower-income participants volunteered for more hours than their wealthier or more educated counterparts (Tang et al., 2010b), while those with poorer health were more likely to discontinue volunteering than their healthier peers (Tang et al., 2010a). A study found that lower-income stipended volunteers had longer retention periods than higher-income non-stipended peers (McBride et al., 2011). During the COVID-19 pandemic, a telephone-based volunteer program also allowed participants to maintain their engagement, which might have otherwise been disrupted (Fields et al., 2023).

## Discussion

In our review of empirical studies that examined formal volunteering among economically marginalized older adults in the U.S., we identified multiple themes related to associated factors and outcomes of volunteering at the individual, community, and societal levels.

Most studies in our review showed that older volunteers with lower economic status experienced improvements in health and well-being. Notably, three studies found that these individuals reported significantly higher gains in self-rated health and perceived well-being compared to their more affluent peers (Kim & Halvorsen, 2021; Morrow-Howell et al., 2009; Tang et al., 2010b). This suggests that older adults with lower economic status may experience greater benefits from volunteering despite being less likely to participate. Given that older adults with lower economic status tend to experience worse health and well-being overall (Choi et al., 2020), volunteering may help mitigate these disparities.

Older volunteers also strengthened nonprofit organizational capacity through sustained participation. Those with lower economic status volunteered more hours than their more affluent peers (Tang et al., 2010b) and maintained their engagement through a telephone-based program during the COVID-19 pandemic (Fields et al., 2023). Despite having fewer resources, they may gain awareness of community or governmental resources—often delivered through the organizations where they serve (Kim & Halvorsen, 2021)—as well as enhanced social connectedness (Hood et al., 2018), empowerment, and a sense of value (Tang et al., 2010b), which may in turn reinforce their continued engagement. These potential mechanisms may reflect the reciprocal nature of volunteer involvement over time, as informed by the ecological framework. In addition, although not directly examined in the included studies, engaging older adults across diverse socioeconomic strata may also serve as a pathway for broader societal benefits—such as greater nonprofit sustainability, enhanced intergenerational solidarity, and strengthened community bonds (Gonzales et al., 2015).

Our findings highlight the importance of facilitating volunteer opportunities for older adults who might benefit but may also be less likely to take part than those with more resources. Modifiable community-level or institutional factors could be especially crucial for older adults with limited resources. Age-friendly community infrastructure, such as accessible public transportation, promoting volunteer opportunities within neighborhoods, and providing affordable senior housing and connected community resources, may reduce access barriers. Institutional practices, including stipends, transportation support, and flexible scheduling, may enhance initial engagement and retention. Cultural competence in organizations, including providing language assistance or hiring staff from diverse backgrounds, can foster inclusivity in volunteer opportunities.

At a broader level, policy support may play a crucial role in fostering the inclusion of economically marginalized older adults in volunteer activities. Several studies recruited participants through federally subsidized programs or national nonprofit initiatives. These programs, supported by public and nonprofit agencies, aim to promote socioeconomic diversity among older volunteers, and policies that expand these programs and create new ones could significantly enhance volunteerism across a broader socioeconomic spectrum.

### Gaps in the Existing Literature and Future Research Directions

In this review, half of the included studies recruited participants from the Senior Companion Program or Experience Corps. Future research should explore other federal or local volunteer programs. For example, similar to the Senior Companion Program, the Foster Grandparents Program and Retired and Senior Volunteer Program (RSVP) are other federally subsidized programs that provide material incentives to volunteers, such as hourly stipends for those meeting income eligibility or reimbursement for transportation, which may encourage participation among older individuals with lower economic status. Additional programs worthy of study include major volunteer programs run by nonprofit organizations that rarely specifically target older volunteers yet may have many in their ranks (e.g., Big Brothers Big Sisters and Habitat for Humanity), as well as the countless local programs organized by smaller nonprofits.

Future research may explore the organizational and policy factors affecting the implementation of these supports. For example, studies could investigate which institutional capacities or funding sources allow organizations to provide stipends, transportation, or translation services. Scholars may examine how organizations navigate competing priorities—such as funding constraints, volunteer retention, and equity goals—when designing inclusive programs.

Across the studies in this review, participants were predominantly female or White and were often recruited from specific geographic regions. Future research may prioritize greater demographic and geographic diversity in participant recruitment. While four studies in this review investigated nationally representative samples from the HRS, researchers might also consider other nationally representative datasets, such as the Midlife in the United States (MIDUS), National Social Life, Health, and Aging Project (NSHAP), Current Population Survey (CPS), and National Survey of Black Americans, which all track volunteering among study participants. These datasets may offer opportunities to explore additional factors or outcomes not captured by the HRS.

While this review focused on economic marginalization, it is crucial to recognize that economic disadvantage rarely occurs in isolation; instead, it is deeply intertwined with other social identities, such as race, ethnicity, immigrant status, and gender. Older people of color and immigrants are more likely to experience economic insecurity, limited educational opportunities, and poor health as a result of cumulative segregation and structural discrimination across the life course, which can restrict their access to volunteer opportunities (Gonzales et al., 2015, 2023). The facilitators identified in this review may indirectly reflect these intersecting disadvantages, which were particularly evident in studies involving predominantly low-income African American, Hispanic, and immigrant older adults, where institutional supports such as stipends, training, transportation assistance, and culturally responsive designs reduced barriers and enhanced accessibility.

Caregiving roles also intersect with economic disadvantage. Older women—who are more likely than men to serve as family caregivers and experience career interruptions related to caregiving—often face time constraints and financial vulnerability in later life (Park & Morrow-Howell, 2020). These caregiving responsibilities could prevent some individuals from volunteering due to limited resources; however, these responsibilities might also increase volunteering due to the desire to help others in need, given their own experiences. Future research may adopt intersectional approaches to understand how these overlapping identities and structural conditions shape not only access to volunteering but also the quality of engagement and perceived benefits.

Although formal volunteering is associated with various health benefits among older adults with low income or wealth, most of the included studies in this review focused on self-reported health measures. There is growing interest in exploring the relationships between formal volunteering and objective health measures, such as epigenetic aging and cardiovascular disease, using biomarker data (e.g., Kim et al., 2023, 2025). Future research could examine these relationships across economic strata.

While existing research has largely focused on individual outcomes, the broader inclusion of socioeconomically diverse older adults in formal volunteering may also benefit communities and society by strengthening social cohesion, reducing reliance on social programs, and supporting the nonprofit sector (Gonzales et al., 2015). Future studies may empirically investigate these community and societal outcomes in relation to formal volunteering among socioeconomically diverse older populations.

Only four qualitative studies and three mixed-methods studies were identified, while the majority of the included studies employed quantitative approaches. Formal volunteering is often examined quantitatively, typically using questions about whether participants engaged in volunteer work during a specified period and the amount of time spent on such activities. However, the reasons participants choose to volunteer and how they experience volunteering are rarely explored. Given the diverse nature of volunteering experiences among individuals, there is a significant opportunity to gain deeper insights and understanding through qualitative and mixed-methods research designs.

### Limitations

Several limitations in this study must be acknowledged. One key limitation of scoping reviews is the emphasis on breadth over depth, prioritizing the range of topics covered rather than the quality of the included studies (Tricco et al., 2018).

Additionally, there exists a body of grey literature encompassing program reports and policy discussions that could offer valuable insights but were not included in this review due to our methodological choice to only consider peer-reviewed empirical studies. Our decision may have excluded some of the most current research in non-academic contexts.

This scoping review was also limited to articles published in or after 2005. Findings may not fully capture the effects of major societal disruptions, such as the 2008 economic recession and the COVID-19 pandemic, which likely affected volunteering patterns among older adults and disproportionately constrained opportunities for socioeconomically disadvantaged individuals.

Furthermore, the definitions of “low income” and “low wealth” varied across studies, using different thresholds and program-based criteria. This limits strict comparability across data sources and may influence which older adults were captured as “economically marginalized.” In addition, half of the studies included in this review focused on participants from two volunteer programs—the Senior Companion Program and the Experience Corps—which may limit the generalizability of findings to other volunteering contexts. The geographic scope was also restricted to studies conducted in the U.S. While this focus was intentional, given the contextual differences across countries, our findings might not be applicable in non-U.S. contexts. Lastly, the studies in this review examined only formal volunteering, which may not represent broader volunteer experiences, such as informal helping or political forms of civic activities.

## Conclusions

Volunteering in later life is widely recognized for its various health and well-being benefits as well as its positive impacts on communities and society. However, less attention has been given to older volunteers with lower income or wealth. This scoping review found that various individual, community, and societal factors are associated with formal volunteering among economically marginalized older adults, as well as their related outcomes. We highlight the importance of ensuring that all older adults, especially those with limited economic resources, have equitable opportunities to participate in and thrive through volunteer engagement.

## Supplemental Material

Supplemental Material - Formal Volunteering Among Economically Marginalized Older Americans: A Scoping Review Supplemental Material for Formal Volunteering Among Economically Marginalized Older Americans: A Scoping Review by Byeongju Ryu, Jihye Baek, Christina Matz, and Cal J. Halvorsen in Journal of Applied Gerontology.
