# Supplementary material for: Formal Volunteering Among Economically Marginalized Older Americans: A Scoping Review
Source: J Appl Gerontol. 2026 Jan 3;45(12):2569–83. doi: 10.1177/07334648251410281 (PMC13740186; doi:10.1177/07334648251410281)
Supplement: Supplementary file 1 [file sj-pdf-1-jag-10.1177_07334648251410281.pdf]

Supplementary Appendix A. Preferred Reporting Items for Systematic Reviews and Meta-Analyses Extension for Scoping Reviews (PRISMA-ScR) checklist

SECTION	ITEM	PRISMA-ScR CHECKLIST ITEM	REPORTED ON PAGE #
**TITLE**			
Title	1	Identify the report as a scoping review.	1
**ABSTRACT**			
Structured summary	2	Provide a structured summary that includes (as applicable): background, objectives, eligibility criteria, sources of evidence, charting methods, results, and conclusions that relate to the review questions and objectives.	2
**INTRODUCTION**			
Rationale	3	Describe the rationale for the review in the context of what is already known. Explain why the review questions/objectives lend themselves to a scoping review approach.	4-5
Objectives	4	Provide an explicit statement of the questions and objectives being addressed with reference to their key elements (e.g., population or participants, concepts, and context) or other relevant key elements used to conceptualize the review questions and/or objectives.	5-6
**METHODS**			
Protocol and registration	5	Indicate whether a review protocol exists; state if and where it can be accessed (e.g., a Web address); and if available, provide registration information, including the registration number.	26
Eligibility criteria	6	Specify characteristics of the sources of evidence used as eligibility criteria (e.g., years considered, language, and publication status), and provide a rationale.	8-9
Information sources*	7	Describe all information sources in the search (e.g., databases with dates of coverage and contact with authors to identify additional sources), as well as the date the most recent search was executed.	7; Figure 2
Search	8	Present the full electronic search strategy for at least 1 database, including any limits used, such that it could be repeated.	7; Table 1
Selection of sources of evidence†	9	State the process for selecting sources of evidence (i.e., screening and eligibility) included in the scoping review.	8-9
Data charting process‡	10	Describe the methods of charting data from the included sources of evidence (e.g., calibrated forms or forms that have been tested by the team before their use, and whether	9; Table 2; Supplementary Appendices B and C

SECTION	ITEM	PRISMA-ScR CHECKLIST ITEM	REPORTED ON PAGE #
		data charting was done independently or in duplicate) and any processes for obtaining and confirming data from investigators.	
Data items	11	List and define all variables for which data were sought and any assumptions and simplifications made.	9-10; Table 2; Supplementary Appendices B and C
Critical appraisal of individual sources of evidence§	12	If done, provide a rationale for conducting a critical appraisal of included sources of evidence; describe the methods used and how this information was used in any data synthesis (if appropriate).	NA; not the objective of this scoping review
Synthesis of results	13	Describe the methods of handling and summarizing the data that were charted.	9-10
**RESULTS**			
Selection of sources of evidence	14	Give numbers of sources of evidence screened, assessed for eligibility, and included in the review, with reasons for exclusions at each stage, ideally using a flow diagram.	Figure 2
Characteristics of sources of evidence	15	For each source of evidence, present characteristics for which data were charted and provide the citations.	Table 2; Supplementary Appendices B and C
Critical appraisal within sources of evidence	16	If done, present data on critical appraisal of included sources of evidence (see item 12).	NA; not the objective of this scoping review
Results of individual sources of evidence	17	For each included source of evidence, present the relevant data that were charted that relate to the review questions and objectives.	10-12; Table 2; Supplementary Appendices B and C
Synthesis of results	18	Summarize and/or present the charting results as they relate to the review questions and objectives.	13-20; Tables 2, 3, and 4; Figure 3; Supplementary Appendices B and C
**DISCUSSION**			
Summary of evidence	19	Summarize the main results (including an overview of concepts, themes, and types of evidence available), link to the review questions and objectives, and consider the relevance to key groups.	20-25
Limitations	20	Discuss the limitations of the scoping review process.	25-26
Conclusions	21	Provide a general interpretation of the results with respect to the review questions and objectives, as well as potential implications and/or next steps.	26
**FUNDING**			
Funding	22	Describe sources of funding for the included sources of evidence, as well as sources of funding for the scoping review. Describe the role of the funders of the scoping review.	NA, no funding for this scoping review

Notes: JBI = Joanna Briggs Institute; PRISMA-ScR = Preferred Reporting Items for Systematic reviews and Meta-Analyses extension for Scoping Reviews.

* Where *sources of evidence* (see second footnote) are compiled from, such as bibliographic databases, social media platforms, and Web sites.
† A more inclusive/heterogeneous term used to account for the different types of evidence or data sources (e.g., quantitative and/or qualitative research, expert opinion, and policy documents) that may be eligible in a scoping review as opposed to only studies. This is not to be confused with *information sources* (see first footnote).
‡ The frameworks by Arksey and O'Malley (6) and Levac and colleagues (7) and the JBI guidance (4, 5) refer to the process of data extraction in a scoping review as data charting.
§ The process of systematically examining research evidence to assess its validity, results, and relevance before using it to inform a decision. This term is used for items 12 and 19 instead of "risk of bias" (which is more applicable to systematic reviews of interventions) to include and acknowledge the various sources of evidence that may be used in a scoping review (e.g., quantitative and/or qualitative research, expert opinion, and policy document).
From: Tricco AC, Lillie E, Zarin W, O'Brien KK, Colquhoun H, Levac D, et al. PRISMA Extension for Scoping Reviews (PRISMAScR): Checklist and Explanation. Ann Intern Med. 2018;169:467–473. https://doi.org/10.7326/M18-0850

Supplementary Appendix B. Study and participants characteristics (N=21)

#	Study	Study Objectives	Participants Characteristics	Study Design (Analytic Approach)	Data	Theoretical or Conceptual Frameworks
1	Butler (2006)	• Understand the meaning of the Senior Companion Program (SCP) from the perspectives of program participants and the larger community<br>• Assess the effectiveness of various measures and open-ended questions for ongoing evaluation of the program	• Volunteers (*n*=34): 79.4% female; mean age 74; 41.2% widowed; 58.8% lived alone; 20.6% less than high school, 64.7% high school, 14.7% more than high school; 100% below 125% of the FPL<br>• Clients (*n*=32): 84.4% female; mean age 82; 78.1% widowed; 84.4% lived alone; 40.6% less than high school, 34.4% high school, 25.0% more than high school; $650.50 monthly median income (40% said they typically ran out of money before the end of the month)	Mixed-method and cross-sectional	Primary (face-to-face interviews)	Continuity theory (Atchely, 1999)<br>Role theory (Adelmann, 1994)
2	Cao et al. (2021)	Investigate the challenges and benefits of volunteering among low-income and culturally and linguistically diverse older adults	*N*=70; 60% female; mean age 76.8; 44.3% White, 34.3% Black, 21.4% Asian; Predominantly low-income (distribution not listed)	Qualitative (Rapid and Rigorous Qualitative Data Analysis; RADaR)	Primary (focus group interviews)	Active aging framework (World Health Organization, 2002)
3	Cao et al. (2023a)	Explore the structures and dynamics of social interactions within a multicultural volunteer program	*N*=83; 61.6% female; mean age 75.9; 52.5% White, 33.8% Black, 12.5% Asian or Pacific Islander; 55.6% more than high school, 27.8% high school, 16.7% less than high school; 45.2% married, 26.2% divorced/separated/never married, 28.6% widowed; Predominantly low-income (distribution not listed)	Convergent mixed-method and cross-sectional (social network analysis and constructive grounded theory approach)	Primary (survey and focus group interviews)	Social capital theory (Claridge, 2018)
4	Cao et al. (2023b)	Examine how older adults' loneliness is correlated with their peers' loneliness within the Senior Companions Program (SCP)	*N*=41; 52.6% female; mean age 77.0; 19.5% Asian or Pacific Islander, 26.8% Black or African American, 51.2% White; 21.6% no high school degree, 16.2% high school degree or equivalent, 21.6% some college no degree, 5.4% associates degree, 13.5% Bachelor's degree, 21.6% graduate or professional; 12.5% divorced or separated, 47.5% married, 12.5% never married, 27.5% widowed; Predominantly low-income (distribution not listed)	Quantitative and cross-sectional (Linear Network Autocorrelation Modelling; LNAM)	Primary (survey)	Social Contagion Theory (Christakis & Fowler, 2013)

#	Study	Study Objectives	Participants Characteristics	Study Design (Analytic Approach)	Data	Theoretical or Conceptual Frameworks
5	Carlson et al. (2009)	Determine if the Experience Corps (EC) program improves age-vulnerable executive functions and increases activity in brain regions	• Volunteers in the EC program (*n*=8): mean age 68; mean education year 12.4; 62% widowed; 100% African American; Predominantly low-income (distribution not listed)<br>• Those waitlisted for enrollment in the EC program (control group; not enrolled in the Experience Corps program; *n*=9): mean age 67.8; mean education year 11.6; 11.1% widowed; 100% African American	Quantitative and pre-and post-test (Repeated measures ANOVA)	Primary (neuroimaging data)	Not provided
6	Cho et al. (2018)	Examine the association between postretirement work and volunteering among retirees<br>Determine whether this association varies across poverty status	*N*=6,663; 54.0% female; mean age 75.4; 75.3% White, 13.5% African-American, 8.8% Hispanic; 58.7% married; mean education years 12.6; 26.9% moderate poverty group (≥185 FPL & <300 FPL), 23.9% near poverty group (≥100 FPL & <185 FPL), 11.7% poverty group (<100 FPL)	Quantitative and cross-sectional (ANOVA; ordered logistic regression)	Secondary (survey; Health and Retirement Study)	Role enhancement theory (Rozario et al., 2004)<br>Role strain theory (Goode, 1960)
7	Cho et al. (2020)	Investigate the association between volunteering and perceptions of usefulness and easiness of information communication and technologies among older adults<br>Assess if this association differs across their income status	*N*=901; 56.2% female; mean age 70.3; 29.5% non-White; 61.9% married; mean education years 12.7; 31.7% below FPL	Quantitative and cross-sectional (Multinomial logistic regression)	Secondary (survey; Health and Retirement Study)	The technology acceptance model (TAM; Guner & Acarturk, 2018)
8	Clark-Ibáñez et al. (2021)	Describe a program, Cultivando Sabiduría (Cultivating Wisdom), created to honor and expand civic engagement in low-income, Spanish-speaking Latinx older adults	*N*=177; age: 55+; 100% Latinx; Predominantly low-income (distribution not listed)	Mixed-method and longitudinal (2017 – 2020; descriptive analysis)	Primary (survey, ethnography, semi-structured interviews, oral histories, photo-elicitation interview, and auto-ethnography)	• Community cultural wealth (Tara Yosso, 2006)<br>• Life course perspective (Stroller & Gibson, 1999; Elder et al., 2003)<br>• Life course developmental theory (Elder, 1998; 2008)

#	Study	Study Objectives	Participants Characteristics	Study Design (Analytic Approach)	Data	Theoretical or Conceptual Frameworks
9	Fields et al. (2023)	Explore the experiences of Senior Companion volunteers in the Caring Callers Program during the COVID-19 pandemic	*N*=18; 100% female; mean age 73.2; 94.4% Non-Hispanic Black, 5.6% Hispanic; 61.1% more than high school degree, 33.3% high school degree, 5.6% less than high school degree; 16.7% married, 83.3% widowed/divorced/never married; Predominantly low-income (distribution not listed)	Qualitative (RADaR: Rigorous and Accelerated Data Reduction)	Primary (semi-structured and individual interviews)	Not provided
10	Hood et al. (2018)	Understand the perceived psychosocial benefits of volunteering among urban-dwelling, low-income older adult women volunteers	*N*=59; 100% female; 49.3% age 65-74, 27.1% age 75 or older, 20.3% age 55-64; 81.4% African American, 17% White; 33.9% more than high school, 49.2% high school, 17% less than high school; Predominantly low-income (62.7% below FPL)	Qualitative (Inductive content analysis)	Primary (focus group interviews)	Not provided
11	Kim & Halvorsen. (2021)	Investigate whether (1) formal volunteering influences self-reported health and the number of depressive symptoms in older adults and (2) how these relationships differ between the highest and lowest wealth quintiles	*N*=90,881 observations (22,958 individuals; 16,163 households); 58.5% female; mean age 69.3; 76.7% White, 16.9% Black, 11.2% Hispanic; mean education years 12.7; 61.8% married/partnered; lowest wealth quintile (*n*=16,912 observations)	Quantitative, longitudinal, and quasi-experimental (2004 through 2016 data; inverse probability of treatment weighting; ordered logistic regression)	Secondary (survey; Health and Retirement Study)	Not provided
12	Martinez et al. (2011)	Explore definitions of volunteering, and barriers and facilitators of volunteering for older adults with lower socio-economic status and diverse ethnic backgrounds	*N*=35; 57.1% female; mean age 74.3; 11.4% married; 62.9% African American, 37.1% White; Around half reported at least 12 years of formal education; 71% reported incomes of less than $15,000 per year	Primarily qualitative (focus group; also incorporated responses from a survey questionnaire about basic demographic and social characteristics)	Primary (focus group interviews and a survey questionnaire)	Not provided
13	McBride et al. (2011)	Assess stipend status relative to volunteer sociodemographic characteristics, motivations, intensity, and duration of time served, and volunteers' self-reported benefits	*N*=263; 89% female; mean age 65; 54% African American, 39% White, 1% Asian, 5% multiracial or other race; mean education years 14 years; mean household income per year $30,000 - $35,000; 36% married/partnered	Quantitative and pre-and post-test (Chi-square analyses, t-tests, and regression with robust standard errors analysis)	Primary (survey)	Not provided

#	Study	Study Objectives	Participants Characteristics	Study Design (Analytic Approach)	Data	Theoretical or Conceptual Frameworks
14	Moon. (2012)	Examine the types and degree of involvement of productive activities among urban, lower-income African American older adults and to explore factors associated with well-being	*N*=531; 75% female; mean age 73; 100% African American; 52.7% had less than a high school education; 51.1% widowed; 79.1% had an annual income less than $20,000 and 36.7% had less than $7,500	Quantitative and cross-sectional (Bivariate associations and multiple regression analysis)	Primary (survey)	Sherraden et al. (2001) model of productivity in later life
15	Morrow-Howell et al. (2009)	Document the benefits of volunteering perceived by older adults and to explain variation in these self-perceived benefits	*N*=401; 65.6% female; age range 51-90 (41.4% were in the age range between 71 and 80); 79.6% White/Caucasian; 47.6% married/partnered; 11.5% currently employed; 79.8% had a college degree or above; More than one quarter earned an annual household income of less than $25,000	Quantitative and cross-sectional (Generalized estimating equations regressions)	Primary (survey)	Developed by the authors for this study through synthesis of previous literature (conceptual framework was unnamed)
16	Park et al. (2017)	Examine the role living in senior housing has on the likelihood of volunteering	*N*=1,415; 77% female; mean age 77.3; 76% White; mean education years 11.6; 100% low income (below 185% FPL)	Quantitative and cross-sectional (propensity score weighting; logistic regression models)	Secondary (survey; Health and Retirement Study)	Person-environment fit perspective<br>Environmental docility hypothesis (Lawton, 1980; Lawton & Nahemow, 1973)
17	Tan et al. (2006)	Report on changes in physical activity levels among older adult participants in the Baltimore Experience Corps program	• Participants in the Experience Corps program (*n*=59): 92% female; 55% between 59 and 70 years old; 83% had a high school degree or less; 24% married; 97% African American; 65% earned income less than $15,000 per year<br>• Those waitlisted for enrollment in the Experience Corps program (control group; not enrolled in the Experience Corps program; *n*=54): 96% female; 66% between 59 and 70 years old; 85% had a high school degree or less; 23% married; 96% African American; 69% earned income less than $15,000 per year	Quantitative and randomized controlled trial (t-test, chi-square test, and multivariate linear regression)	Primary (survey)	The social model for health promotion (Fried et al., 2004)

#	Study	Study Objectives	Participants Characteristics	Study Design (Analytic Approach)	Data	Theoretical or Conceptual Frameworks
18	Tan et al. (2009)	Determine if there were sustained increases in physical activity with participation in the Baltimore Experience Corps program	• Participants in the Experience Corps program (*n*=71): 100% female; mean age 71.2; 86.9% had a high school degree or less; 14.1% married; 100% African American; 60.5% earned income less than $15,000 per year<br>• Participants in the Baltimore Women's Health and Aging Study (comparison group; not enrolled in the Experience Corps program; *n*=154): 100% female; mean age 73.0; 84.7% had a high school degree or less; 22.7% married; 100% African American; 53.4% earned income less than $15,000 per year	Quantitative and longitudinal (36 months of follow-up; propensity score method and regression analysis)	Primary (survey)	The social model for health promotion (Fried et al., 2004)
19	Tang et al. (2009)	Describe, from the older volunteers' perspectives, the importance of institutional facilitation of volunteering and to examine if the perceived importance of institutional facilitation varies by socioeconomic demographics	*N*=374; 64.7% female; mean age 72.8; 80.0% White; 47.3% married; mean education years 15.7; 9.9% employed; median family income was between $40,000 and $59,999, with 10.4% having $80,000 or more; 47.9% had an annual family income less than $40,000, with 11.0% having less than $15,000	Quantitative and cross-sectional (multiple analysis of covariance; MANCOVA)	Primary (survey)	Institutional theory (Beverly & Sherraden, 1999; Neale, 1987)
20	Tang et al. (2010a)	Evaluate the individual and program characteristics that influence the turnover of older volunteers	*N*=207; 66.7% female; mean age 73; 74.9% White; 42.5% married; 13.5% currently employed; mean educational years 15.9; About one-quarter had an annual household income of more than $60,000	Quantitative and longitudinal (generalized estimating equations logistic regression analysis)	Primary (survey)	Institutional perspective (Sherraden et al., 2001)
21	Tang et al. (2010b)	Test a theoretical model of volunteering benefits and examine the mechanism through which volunteering benefits older adults	*N*=207; 67% female; mean age 72; 74.6% White; 43.8% married; 12.4% currently employed; mean educational years 15.86; average annual household income close to the range $30,000-$39,000	Quantitative and longitudinal (structured equation modeling)	Primary (survey)	• Institutional perspective (Sherraden et al., 2001)<br>• Socioemotional selectivity theory (Carstensen, 2006; Carstensen & Lockenhoff, 2003)

Note: FPL = federal poverty line

Supplementary Appendix C. Program descriptions, associated factors, and outcomes of formal volunteering among economically marginalized older adults (N=21)

#	Study	Description of Volunteer Program [a]	Associated Factors [b]	Outcomes [b]
1	Butler (2006)	Senior Companion Program (SCP)<br>• Connects older volunteers with frail older adults needing companionship and assistance to stay in their homes<br>• Adults 60 years of age and older are eligible to be SCP volunteers<br>• Receives a stipend ($2.65/hour) and mileage reimbursement for eligible low-income older volunteers<br>• Federally subsidized volunteering program<br>• Location: A rural county in Maine	*Facilitators*<br>Stipend (important for volunteers in meeting their basic needs)<br><br>*Barriers*<br>(Not the focus of this study)	*Volunteers' perspectives*<br>• Benefits: Allowing them to remain actively engaged in their communities; Being able to help neighbors who were in need due to disability, loneliness, or both; Contributed to the volunteers' own sense of well-being<br>• Disadvantages or things that need to be improved: Losing a client or watching their clients in pain; Having a client who has a challenge that volunteers cannot help with; Finding balance (i.e., pushing themselves too hard in their volunteer role to the detriment of their own health and well-being such as having a volunteer role outside home in addition to a family caregiving role at home)<br><br>*Clients' perspectives*<br>• Benefits: Increased social support, decreased isolation, and relieved anxieties about how certain tasks of their basic daily living could be accomplished<br>• Disadvantages or things that need to be improved: Flexibility in scheduling friendly visits
2	Cao et al. (2021)	Senior Companion Program (SCP)<br>• Connects older volunteers with frail older adults needing companionship and transportation support for 15 hours or more each week<br>• Provides monthly in-service training<br>• Receives a stipend ($2.65/hour) and mileage reimbursement for eligible low-income older volunteers<br>• Federally subsidized volunteering program<br>• Location: A Midwest metropolitan area	*Facilitators*<br>(Not the focus of this study)<br><br>*Barriers*<br>• Transportation (lack of transportation from an ethnic enclave; lack of support for car maintenance and limited mileage reimbursement; long process of learning to drive)<br>• Feeling overwhelmed because there were always emergencies in the neighborhood (hard to say no whenever neighbors asked for help; some clients took services for granted and had limited consideration for volunteers' challenges)<br>• Lack of community resources/services and information for volunteers who take care of family members with high functional impairment and care needs	• Stress-relieving benefits: Playing with the clients (e.g., talking and games); Sharing similar cultural and language backgrounds<br>• Training and information benefits: Monthly in-service training about resources (e.g., transportation, community resources)<br>• Peer support and social benefits (e.g., connected with people with the same language and shared culture; carpooling among volunteers)

#	Study	Description of Volunteer Program [a]	Associated Factors [b]	Outcomes [b]
3	Cao et al. (2023a)	Senior Companion Program (SCP; see Cao et al., 2021 for description)	*Facilitators*<br>For cross-cultural volunteering: Intentional facilitations, resources, and organizational commitment for facilitating cross-cultural volunteering (e.g., translation or interpretation services, and orientation, training sessions, or organizational events in a multicultural environment where volunteers from diverse backgrounds gather and interact with each other)<br><br>*Barriers*<br>For cross-cultural volunteering: Preferences for socializing with those from the same gender and country who spoke the same language (i.e., homophily)	Expanded and strengthened social networks through volunteering (i.e., developing friendships with other volunteers and deepening relationships with clients)<br>• Between volunteers: Offered opportunities to interact with volunteers of various demographic and socioeconomic backgrounds through SCP events such as orientation and monthly in-service training (i.e., social bridging)<br>• Between volunteers and clients: Facilitated a sense of purposeful life (i.e., making a difference in clients' lives positively made volunteering meaningful); Reciprocated relationship (i.e., volunteers helped clients navigate the service system; volunteers gained a sense of satisfaction that they are needed and welcomed by clients)
4	Cao et al. (2023b)	• Senior Companion Program (SCP; see Cao et al., 2021 for description)	*Facilitators*<br>(Not the focus of this study)<br><br>*Barriers*<br>(Not the focus of this study)	Preventing the negative reinforcement of loneliness: Less lonely volunteers tended to make friends with lonelier volunteers (In previous research on the general population outside of volunteering contexts, lonely individuals tended to interact with other lonely individuals in the network, reinforcing loneliness through peer associations)
5	Carlson et al. (2009)	Experience Corps (EC) program:<br>• Train and place volunteers in elementary schools for an academic year to assist kindergarten through third-grade teachers with literacy and library functions.<br>• Location: Baltimore, MD	*Facilitators*<br>(Not the focus of this study)<br><br>*Barriers*<br>(Not the focus of this study)	Improved executive functions and increased activity in brain regions (Increased brain activity in the left prefrontal cortex and anterior cingulate cortex over the 6-month interval relative to matched controls), implying the program's potential to reverse cognitive and corresponding neural declines with age
6	Cho et al. (2018)	Not provided with a specific program description (Respondents had volunteered in the past 12 months for religious, educational, health-related, or other charitable organizations)	*Facilitators*<br>Post-retirement work (i.e., paid work after retirement); In the near-poverty group, retirees who engaged in postretirement work spent significantly greater time volunteering than those who did not work after retirement<br><br>*Barriers*<br>(Not the focus of this study)	(Not the focus of this study)

#	Study	Description of Volunteer Program [a]	Associated Factors [b]	Outcomes [b]
7	Cho et al. (2020)	Not provided with a specific program description (Respondents had volunteered in the past 12 months for religious, educational, health-related, or other charitable organizations)	*Facilitators*<br>High perception of the usefulness and easiness of technology significantly increased the likelihood of volunteering among low-income older adults<br><br>*Barriers*<br>(Not the focus of this study)	(Not the focus of this study)
8	Clark-Ibáñez et al. (2021)	Cultivando Sabiduría<br>• Facilitates volunteering in civic affairs (e.g., assistance to teachers and students at school, facilitation of study groups and provision of childcare at church, distribution of food in mobile pantries, and collecting trash in the neighborhoods)<br>• A weekly 3-hour class using the best practices for organizing a civic and culturally appropriate education curriculum<br>• Location: San Diego County, CA	*Facilitators*<br>• Linguistically and culturally relevant program and curriculum (e.g., having the majority of the students, volunteers, and staff be Latinx and bilingual)<br>• Desire to help others, make their communities a better place, and learn a new skill<br><br>*Barriers*<br>Limited English proficiency	• Satisfaction with their new roles as a participant in the program<br>• Learn a new skill (e.g., art, writing, and language)<br>• Respite from caretaking that needs intensive spousal and/or family commitments: Distraction away from their responsibilities at home by learning new things through the program<br>• Socially connected with people outside home

#	Study	Description of Volunteer Program [a]	Associated Factors [b]	Outcomes [b]
9	Fields et al. (2023)	The Caring Callers Program<br>• A telephone-based intervention aims to have older adult peer volunteers provide friendly weekly telephone calls to socially isolated older adults while also connecting them with community resources.<br>• Consists of trained volunteers from the Senior Companion program, a federally subsidized volunteering program for older adults in the U.S.<br>• Location: North Texas	*Facilitators*<br>• Personal values for helping others: Often described the program closely aligned with their perceived self-identity which sparked their interest in participation.<br>• Being well-matched with their clients (i.e., sharing similar interests, similar life experiences) were observed to report greater satisfaction with the program.<br><br>*Barriers*<br>(Not the focus of this study)	• Reciprocity "We were helping each other" (i.e., mutually beneficial to themselves and their clients): Offsetting feelings of isolation and loneliness associated with social distancing in the COVID-19 through social engagement<br>• Purposeful use of time "It gave me something to do": Facilitating responsibility associated with making the phone calls; Sense of identity by fulfilling their value for civic duty; Alleviating the loss of role and identity as a volunteer in COVID-19<br>• Learning new skills "I learned to just listen": Gaining new skills and knowledge of conversations with new people (i.e., clients); Revisiting skills they had but had not utilized for many years; Skill building<br>• Gaining a new perspective "It taught me something too": Applying lessons learned from the conversation with clients to their own lives<br>• Increased feelings of confidence from making weekly calls and expressed interest in continuing as a Caring Callers volunteer<br>• Allowed for volunteers to receive training remotely and opportunity to continue in their volunteer endeavors which may otherwise have been missed due to the pandemic; Telephone-based reassurance programs such as Caring Callers may eliminate barriers to volunteering for older adults limited by mobility, geography, and resources required for face-to-face interactions
10	Hood et al. (2018)	Senior Companion Program<br>• Provides companionship visits and transportation support to their clients for 15 hours or more each week<br>• Receives a stipend ($2.65/hour) and mileage reimbursement for eligible low-income older volunteers<br>• Location: Marion County, IN (central Indiana's most populated urban county)	*Facilitators*<br>(Not the focus of this study)<br><br>*Barriers*<br>(Not the focus of this study)	• Reciprocity and mutual benefit (e.g., clients are also checking on volunteers during the friendly visits)<br>• Reducing social isolation (e.g., opportunity to leave their homes, increased social interaction, facilitated meaningful and friendship relationships beyond the relationship between volunteers and clients)<br>• Improved quality of life (e.g., opportunity to stay active by participating in various activities, improved emotional well-being)<br>• Facilitated a sense of purpose, a sense of accomplishment, and feeling needed (to their clients)<br>• Increased understanding of aging based on their client's experiences (volunteer experiences have better prepared them for their aging process)

#	Study	Description of Volunteer Program [a]	Associated Factors [b]	Outcomes [b]
11	Kim & Halvorsen. (2021)	Not provided with a specific program description (Respondents had volunteered in the past 12 months for religious, educational, health-related, or other charitable organizations)	*Facilitators*<br>(Not the focus of this study)<br><br>*Barriers*<br>(Not the focus of this study)	• Increased self-reported health: The lowest wealth quintile experienced more gains in self-reported health from volunteering than those in the highest wealth quintile group.<br>• Fewer depressive symptoms regardless of wealth status
12	Martinez et al. (2011)	Not provided with a specific program description (Respondents were asked whether they have ever volunteered and what type of volunteer activity they have done).	*Facilitators*<br>• Flexible and reasonable time commitments for volunteer roles (e.g., greater flexibility in volunteer roles can help to stimulate more voluntary activity and maintain current levels)<br>• Better treatment by staff in volunteer settings and acknowledgement of the contributions of volunteers (e.g., reward, certificate, or something that volunteers can use that would be good on their record if they wanted to get employment)<br>• Transportation accessibility (e.g., free parking passes for those who drive, transportation from volunteers' residences to the activity sites, and the setting of activities in accessible locations to eliminate the need for older adults to travel)<br>• Asking for volunteer help, and publicizing volunteering opportunities in a timely fashion<br><br>*Barriers*<br>• Health and medical problems (e.g., limited mobility, loss of vision, and lack of energy with increased age)<br>• Inadequate personal resources (e.g., money and time)<br>• Prior negative experiences (e.g., treatment in the volunteering organization such as not being valued and sometimes being bossed around)<br>• Concern over committing to roles that are too structured (i.e., being obliged to fit into structured activities or schedules, feeling too much like work due to the fixed schedule and lack of flexibility)<br>• Transportation accessibility (e.g., having difficulty getting on and off buses, inaccessible bus routes, and safety concerns for night-time activities)	• Feeling better and a sense of satisfaction ("helping others makes one feel better and gives one a sense of satisfaction")<br>• Keeping alert and active<br>• An opportunity for social interaction

#	Study	Description of Volunteer Program [a]	Associated Factors [b]	Outcomes [b]
13	McBride et al. (2011)	Experience Corps (EC) program<br>• See Carlson et al., 2009, for description<br>• Locations: 18 program sites throughout the U.S.	*Facilitators*<br>• Stipend (e.g., leveraging inclusion of ethnic minorities and those of low income, increasing volunteer retention, and perceiving higher benefits than non-stipended volunteers)<br><br>*Barriers*<br>(Not the focus of this study)	• Compared to non-stipended volunteers, stipended volunteers, who tend to earn less income, reported more perceived benefits than non-stipended volunteers, such as using their time more productively, increasing their number of friends and acquaintances, and being more socially active because of their involvement with the Experience Corps program.<br>• Volunteer organizations could have a longer period of volunteer retention among stipend volunteers.
14	Moon. (2012)	Not provided with a specific program description (Respondents were asked whether they work at an agency, hospital, or church or care for any family member, friend, or neighbor and what types of volunteer work they do).	*Facilitators*<br>(Not the focus of this study)<br><br>*Barriers*<br>(Not the focus of this study)	The effects of level of involvement in volunteering did not predict well-being among the lower-income African American older adults, controlling for sociodemographic and individual capacity factors (e.g., income, physical health, neighborhood satisfaction, and social support during the past 12 months)
15	Morrow-Howell et al. (2009)	13 volunteer programs (e.g., Senior Medicare Patrol, Computer Buddies, and Tax Counseling for the Elderly)<br>• Volunteer activities included teaching, tutoring, assisting with instrumental activities, mentoring, policing/public safety activities, counseling/technical advice, conservation activities, and supportive counselling.<br>• Most programs were run by public agencies (police departments, city and state governments), a few were operated by nonprofit agencies, and one was sponsored by a religious organization. One was a Senior Companion program funded by the federal government	*Facilitators*<br>(Not the focus of this study)<br><br>*Barriers*<br>(Not the focus of this study)	• Older volunteers with lower incomes reported more perceived benefits from volunteering; Perceived benefits were operationalized as a summed score of 11 closed-ended questions across various domains (e.g., contribution, meaningful activity, improved life, socialization, self-worth, personal growth, and health).<br>• Lower-income and lower-educated persons participating in instrumental service programs experienced less benefit than those participating in skilled/technical service programs (i.e., Lower-income and less-educated people felt more empowered or valued doing volunteer tasks that highlighted expertise rather than tasks that relied on less skill, such as meal preparation or transportation).

#	Study	Description of Volunteer Program [a]	Associated Factors [b]	Outcomes [b]
16	Park et al. (2017)	Not provided with a specific program description (Respondents had volunteered in the past 12 months for religious, educational, health-related, or other charitable organizations)	*Facilitators*<br>Living in senior housing (i.e., congregate setting where health and social services are provided to facilitate the residents' aging-in-place) positively affected engagement in formal volunteering after adjusting for personal and environmental factors.<br><br>*Barriers*<br>(Not the focus of this study)	Not applicable (not the focus of this study)
17	Tan et al. (2006)	Experience Corps (EC) program:<br>• See Carlson et al., 2009, for description<br>• Location: Baltimore, MD	*Facilitators*<br>(Not the focus of this study)<br><br>*Barriers*<br>(Not the focus of this study)	Increased physical activity levels at follow-up after 4-8 months (i.e., mean minutes per week and mean kilocalories per week), particularly among those who, prior to service, had low levels of physical activity in a short term
18	Tan et al. (2009)	Experience Corps (EC) program:<br>• See Carlson et al., 2009, for description<br>• Location: Baltimore, MD	*Facilitators*<br>(Not the focus of this study)<br><br>*Barriers*<br>(Not the focus of this study)	The long-term participants (three years of volunteering) in the Experience Corps program reported a sustained increase in physical activity (more kilocalories per week expended through physical activities) as compared with the comparison cohort.
19	Tang et al. (2009)	Older volunteers across 13 volunteer programs in nine states in the U.S. were included in the analysis<br>• Volunteer programs included in this study were sponsored by local, state, and national funding sources.<br>• Older volunteers provided a range of services, including advocacy, education, social services, counseling, transportation, legal services, mentoring, companionship, and administrative work.	*Facilitators*<br>• Low-income and non-White volunteers gave more importance to institutional facilitators of engagement in volunteering; Particularly, low-income was related to a higher level of importance on the provision of compensation in volunteer programs.<br>• Flexibility (e.g., choice of activities and ability to set own schedule)<br><br>*Barriers*<br>(Not the focus of this study)	Not applicable (not the focus of this study)
20	Tang et al. (2010a)	Older volunteers across 10 volunteer programs in the U.S. were included in the analysis	*Facilitators*<br>(Not the focus of this study)<br><br>*Barriers*<br>(Not the focus of this study)	Volunteer turnover (i.e., Lower income and poorer health volunteers were more likely to stop volunteering)

#	Study	Description of Volunteer Program [a]	Associated Factors [b]	Outcomes [b]
21	Tang et al. (2010b)	Older volunteers across 10 volunteer programs in the U.S. were included in the analysis	*Facilitators*<br>• Organizational support (e.g., adequate training, ongoing support, and greater flexibility in choosing activities and schedules) was associated with more hours of volunteering per week.<br><br>*Barriers*<br>(Not the focus of this study)	• Older volunteers with lower socioeconomic status perceived more personal benefits than their counterparts with higher socioeconomic status; Perceived benefits were measured with 10 indicators of socioemotional benefits (e.g., contribution to others, self-esteem, personal growth, meaningful engagement, socialization, and life satisfaction).<br>• Volunteers with lower socioeconomic status committed more hours than their counterparts with higher socioeconomic status.

Notes: [a] Volunteer program descriptions were taken from the manuscripts and reflect the time they were published. Program details may have changed since publication. For example, the age minimum for the Senior Companion Program is now 55 (45 C.F.R. § 2551, 2024). [b] Only associated factors and outcomes explicitly mentioned as relevant to economically marginalized older populations are included in the table. For example, associated factors or outcomes examined among a broader older population without distinguishing by economic status were not included.
